# 

**Published:** 1897-05

**Authors:** 


Vol. XVIII. MAY, 1897. No. 5.
PUBLISHED MONTHLY AT $3 A YEAR. SINGLE COPIES, 30 CENTS.

LIBRARY
Indexed
S. G. O.

### THE JOURNAL OF COMPARATIVE MEDICINE AND VETERINARY ARCHIVES

EDITED AND PUBLISHED BY
RUSH SHIPPEN HUIDEKOPER, Veterinarian (Alfort),
W. HORACE HOSKINS, D.V.S.,
H. D. GILL, V.S.

### UNITED STATES VETERINARY MEDICAL ASSOCIATION.

**Proceedings of the Thirty-third Annual Meeting held at Buffalo, N. Y., September, 1896. (Continued.)**

A Study of Cathartics (*concluded*). By M. H. REYNOLDS, M.D., V.M. . . . 177
Physiological Variations. By W. L. WILLIAMS, V.S. . . . 192

ALL COMMUNICATIONS AND PAYMENTS SHOULD BE ADDRESSED TO
**Dr. W. Horace Hoskins, Managing Editor, 3452 Ludlow St., Philadelphia, Pa.**
Copies may be had on order of all news-dealers. In London of Baillière, Tindall & Cox.

